# Enhancement of mycobacterial pathogenesis by host interferon-γ

**DOI:** 10.1007/s00018-024-05425-7

**Published:** 2024-09-02

**Authors:** Huynh Tan Hop, Pao-Chi Liao, Hsin-Yi Wu

**Affiliations:** 1 University Center for Bioscience and Biotechnology, National Cheng Kung University, Tainan, 70101 Taiwan; 2 Department of Environmental and Occupational Health, College of Medicine, National Cheng Kung University, Tainan, 70101 Taiwan; 3 Instrumentation Center, National Taiwan University, Taipei, 106 Taiwan

**Keywords:** Macrophages, Pathogenic mycobacteria, Extracellular vesicles, PpsB, AmiD

## Abstract

The cytokine IFNγ is a principal effector of macrophage activation and immune resistance to mycobacterial infection; however, pathogenic mycobacteria are capable of surviving in IFNγ-activated macrophages by largely unknown mechanisms. In this study, we find that pathogenic mycobacteria, including *M. bovis* BCG and *M. tuberculosis* can sense IFNγ to promote their proliferative activity and virulence phenotype. Moreover, interaction with the host intracellular environment increases the susceptibility of mycobacteria to IFNγ through upregulating expression of *mmpL10*, a mycobacterial IFNγ receptor, thereby facilitating IFNγ-dependent survival and growth of mycobacteria in macrophages. Transmission electron microscopy analysis reveals that IFNγ triggers the secretion of extracellular vesicles, an essential virulence strategy of intracellular mycobacteria, while proteomics identifies numerous pivotal IFNγ-induced effectors required for mycobacterial infection in macrophages. Our study suggests that sensing host IFNγ is a crucial virulence mechanism used by pathogenic mycobacteria to survive and proliferate inside macrophages.

**Supplementary Information:**

The online version contains supplementary material available at 10.1007/s00018-024-05425-7.

## Introduction

Mycobacterium tuberculosis is arguably the most successful pathogen on earth and continues to cause an estimated 1.5 million casualties annually. A critical virulence strategy relies on the survival of mycobacteria within host macrophages [11]. Macrophage cells are essential immune effectors at the front line of host defense against mycobacterial infection. They express receptors that allow them to uptake and degrade pathogens using an array of different effector mechanisms, followed by activating the immune system through a so-called cross-presentation [9]. However, pathogenic mycobacteria can circumvent and dampen many host anti-mycobacterial effects, primarily by as-yet-unknown mechanisms, thereby establishing a niche to survive and proliferate inside the host cells [1].

Cytokines are essential for the immune response of macrophages to mycobacterial infection. Each cytokine binds to a specific surface receptor to activate downstream signaling cascade(s) that may enhance or attenuate anti-mycobacterial effects in macrophages [3, 13]. However, numerous pathogenic bacteria such as *Escherichia coli*, *Shigella flexneri*, *Pseudomonas aeruginosa* and *Neisseria meningitidis* have been found to directly sense host cytokines to facilitate their invasion and survival [28, 30, 39, 65], suggesting that sensing of host cytokines may be an evolutionarily conserved virulence mechanism in pathogenic bacteria that recognizes host immune activation.

Intriguingly, Ahmed et al. recently uncovered that *M. tuberculosis* can interact with IFNγ via its membrane protein MmpL10 to augment its fitness; however, interaction with IFNγ also increases the sensitivity of *M. tuberculosis* to isoniazid, an antibiotic indicated in the first-line treatment of TB [2]. Interferon-γ is a key immune effector activating the anti-microbial effect in antigen-presenting cells and protective-immune responses against mycobacterial infection. An important macrophage activation occurs via IFNγ, thereby converting naïve macrophages, in which mycobacteria can survive, into cells that readily destroy the incoming mycobacteria [20, 29], suggesting that recognition of IFNγ may be an unknown virulence mechanism of intracellular mycobacteria to recognize macrophage activation. Therefore, this study is aimed at investigating the molecular changes in pathogenic mycobacteria triggered by IFNγ and how they impact the mycobacteria-macrophage interactions.

## Results

### Direct interaction of IFNγ with M. Bovis BCG

**Fig. 1:**
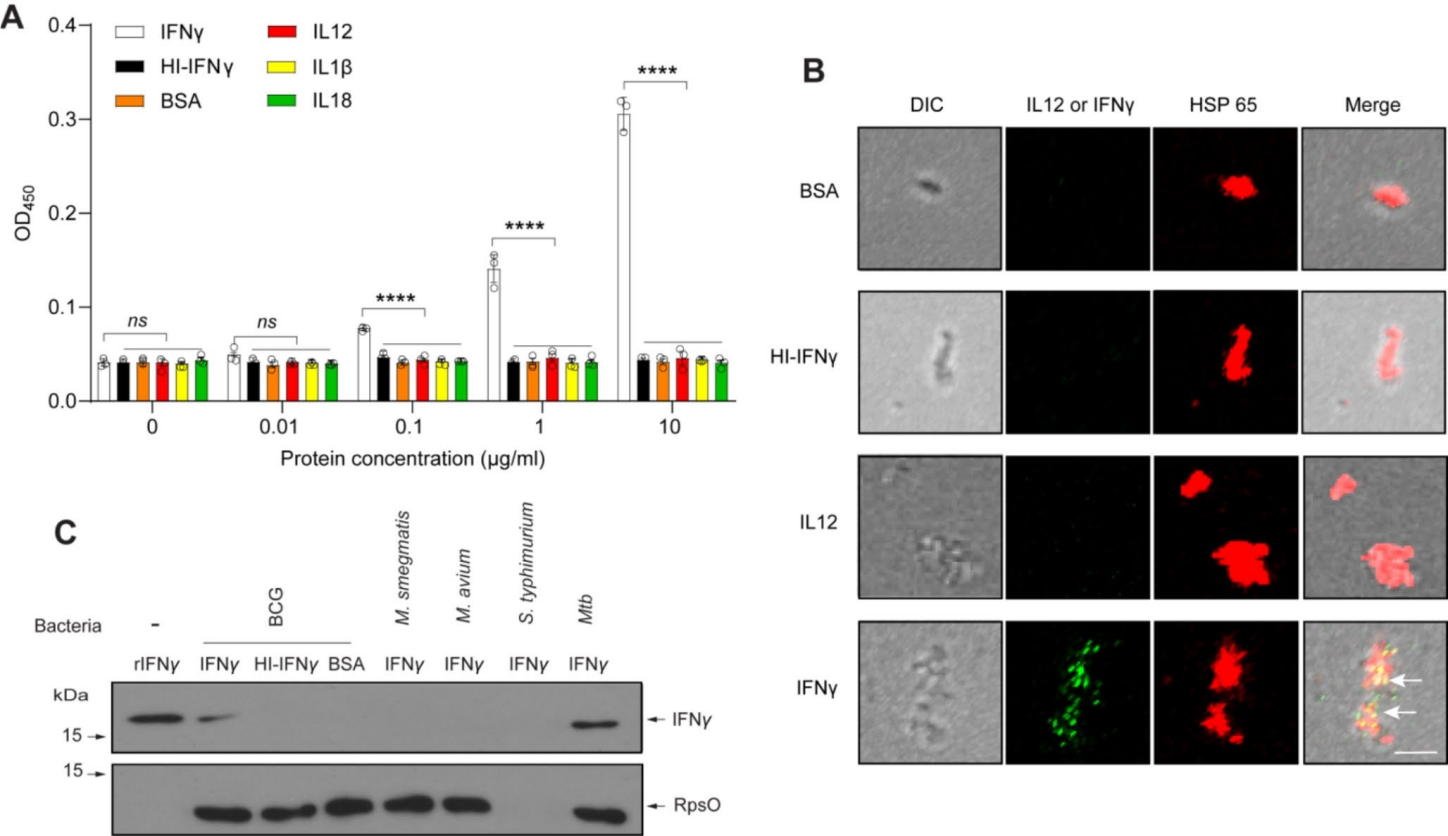
Direct interaction of *M. bovis* BCG and *M. tuberculosis* with interferon-γ. (**A**) Specific binding of IFNγ to *M. bovis* BCG in a dose-dependent manner. Mycobacteria were fixed, incubated with different concentrations of IFNγ, heat inactivated IFNγ (HI-IFNγ), BSA, IL12, IL1β, and IL18, and analyzed by ELISA. (**B**) Immunofluorescence demonstrated IFNγ binding to *M. bovis* BCG. Mycobacteria were fixed, blocked, incubated with 10 µg/ml BSA or cytokines, immunostained, and analyzed by fluorescence microscopy. Scale bar, 1 μm. Arrow indicates protein colocalization. (**C**) Immunoblotting analysis demonstrated IFNγ binding to *M*. *bovis* BCG and *M. tuberculosis*. Bacteria were fixed, blocked, incubated with 10 µg/ml BSA or IFNγ, washed and analyzed by immunoblotting with antibodies against IFNγ and mycobacterial RpsO. Recombinant mouse IFNγ (10 ng) was used as a positive control. Data information: Statistical analysis in Fig. 1A was performed with two-way ANOVA followed by multiple comparisons among groups. The results are the mean values ± standard deviations of three biological replicates each with three technical replicates. *, *p* < 0.05; **, *p* < 0.01; ***, *p* < 0.001; ****, *p* < 0.0001; *ns*, not significant

Since the sequence of the *mmpl10* gene in *M. bovis* BCG is identical to that in *M. tuberculosis* H37Rv, we tested whether IFNγ can also bind to *M. bovis* BCG. First, *M. bovis* BCG was fixed, incubated with different concentrations of IFNγ (active or heat-inactivated form), BSA or other cytokines including interleukin-12 (IL12), interleukin-1β (IL1β), and interleukin-18 (IL18), and analyzed by ELISA. Interferon-γ was found to bind to *M. bovis* BCG in a dose-dependent manner, whereas thermally inactive IFNγ, BSA and other cytokines did not bind to the bacilli (Fig. 1A). To independently analyze the binding of IFNγ to *M. bovis* BCG, we fixed mycobacteria, then incubated with IFNγ (active or heat-inactivated form), IL12 or BSA, immunostained with antibodies against mycobacterial Hsp65, IFNγ or IL12, and analyzed by fluorescence *microscopy*. We found that IFNγ, but not its inactive form, IL12 or BSA, binds to mycobacterial cells (Fig. 1B).

To further validate the interaction of IFNγ with *M. bovis* BCG, we incubated IFNγ with different fixed bacterial strains (*M. bovis* BCG, *M. tuberculosis*, *M. smegmatis*, *M. avium*, and *S. typhimurium*), washed and analyzed by immunoblotting using antibodies against mycobacterial RpsO or IFNγ. Interferon-γ was detected only in the *M. bovis* BCG and *M. tuberculosis* lysates, confirming the binding and retention of IFNγ on the surface of *M. bovis* BCG and *M. tuberculosis* (Fig. 1C). These findings indicate that IFNγ specifically interact with *M. bovis* BCG and *M. tuberculosis*, which belong to the *M. tuberculosis* complex (MTC); therefore, we used *M. bovis* BCG as a model to investigate the interaction of IFNγ with MTC because of its experimental advantages in the laboratory.

### Interferon-γ modulates translational profiles in mycobacteria

**Fig. 2:**
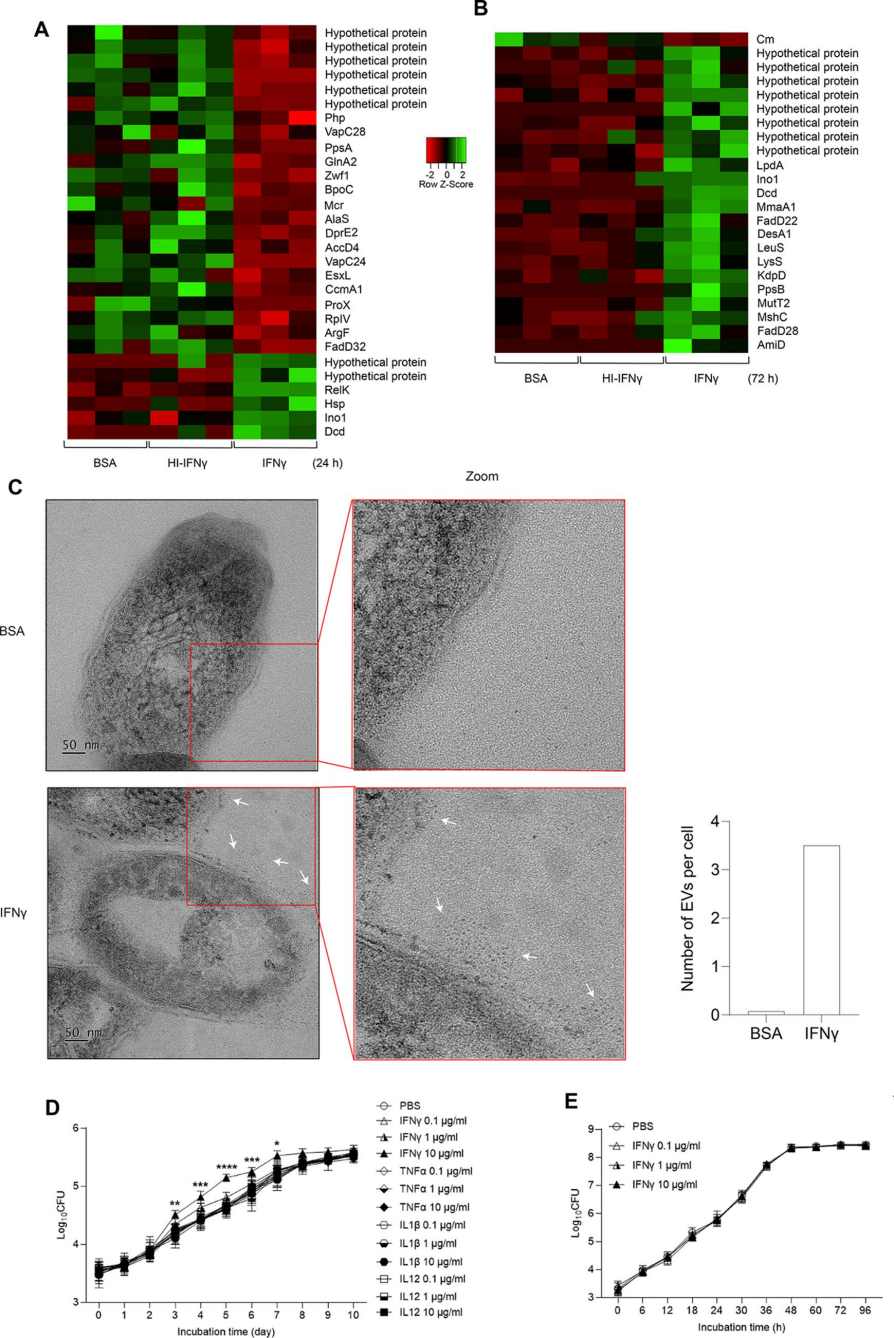
Interferon-γ drives mycobacteria to a virulent phenotype. (**A** and **B**) Interferon-γ modulates mycobacterial proteome profile. *M. bovis* BCG was cultured in 7H9 medium supplemented with 10 µg/ml BSA, IFNγ or HI-IFNγ. Mycobacterial protein samples were extracted at 24 and 72 h post-treatment and analyzed using label free LC-MS/MS. Heatmaps were used to present the most changed proteins in mycobacteria after 24 (**A**) and 72 (**B**) hr of IFNγ treatment. (**C**) Interferon-γ induces the secretion of extracellular vesicles (E.V.s) in mycobacteria. Left panel, *M. bovis* BCG were cultured in 7H9 medium supplemented with 10 µg/ml BSA or IFNγ. At 72 h post-treatment, bacteria were collected and analyzed by transmission electron microscopy. Righ panel, quantification of EVs released by *M. bovis* BCG treated with BSA or IFNγ. (**D**) Interferon-γ induces mycobacterial growth in culture. *M. bovis* BCG was cultured in 7H9 broth supplemented with different concentrations of cytokines. At the indicated times, the inoculum was serially diluted in PBS, and plated on 7H10 agar plates. Bacterial CFU was determined after 3–4 weeks of incubation at 37^o^C. (**E**) Growth of *M. smegmatis* in the presence of IFNγ. Mycobacteria were cultured in 7H9 broth supplemented with 0.1, 1–10 µg/ml IFNγ. At the indicated times, the inoculum was serially diluted in PBS, and plated on 7H10 agar plates. The bacterial CFU was determined after 4–6 days of incubation at 37^o^C. Data information: Statistical analyses in Fig. 2D and E were performed with two-way ANOVA followed by multiple comparisons among groups. The results are the mean values ± standard deviations of three biological replicates each with three technical replicates. *, *p* < 0.05; **, *p* < 0.01; ***, *p* < 0.001; ****, *p* < 0.0001; *ns*, not significant

To dissect the interaction of IFNγ and pathogenic mycobacteria, *M. bovis* BCG was treated with IFNγ, heat-inactivated IFNγ, or BSA, followed by comparative proteomic analysis at 24 and 72 h after treatment (Supplemental Dataset 1). The most changed proteins in IFNγ-treated mycobacteria compared to BSA- and inactivated IFNγ-treated mycobacteria were listed in Table 1; at 24 h post-treatment, 23 of 29 (approximately 79.3%) most changed proteins are the reduction while 22 of 23 (approximately 95.6%) most changed proteins were the induction at 72 h post-treatment. The inhibited proteins by IFNγ at 24 h are enzymes involved in different cellular processes, including toxin-antitoxin system, pentose phosphate pathway, glutamine, and lipid metabolism (Fig. 2A). Some of the inhibited proteins such as ribonuclease VapC28, probable alpha-methylacyl-CoA racemase Mcr, ESAT-6-like protein EsxL and possible invasion protein are negative regulators of mycobacterial growth [15, 51], suggesting the inhibition of these proteins by IFNγ may provide growth advantage for mycobacteria. Notably, a marked inhibition of glucose-6-phosphate 1-dehydrogenase Zwf1, a crucial enzyme of first reaction in the pentose phosphate pathway, suggests interferon-γ may trigger carbon metabolism remodeling, an underlying mechanism to survive inside the host cells and to develop drug tolerance and resistance of mycobacteria [18, 41]. In addition, elevated expression of toxin RelK, heat shock protein Hsp, and myo-inositol-1-phosphate synthase Ino1 may benefit mycobacteria in response to oxidative and heat stresses, antibiotic treatment and host defenses [25, 32, 58].

**Table 1:**
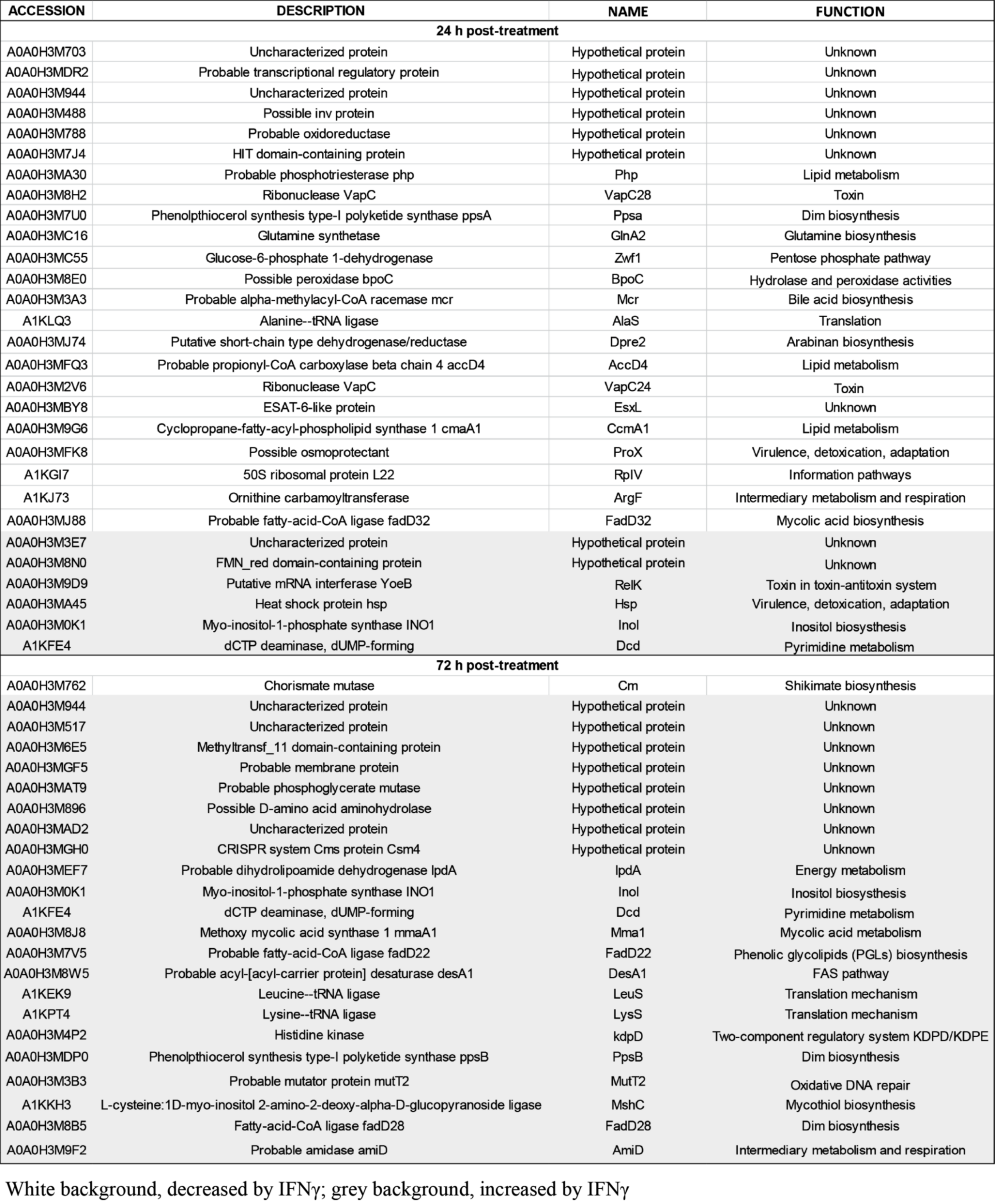
The most regulated proteins in *M. Bovis* BCG at 24 and 72 h after IFNγ treatment

Furthermore, prolonged treatment of IFNγ promotes mycobacteria to a virulence phenotype; a variety of growth effectors and major virulence factors were elevated in IFNγ-treated mycobacteria compared to BSA- and heat-inactivated IFNγ-treated cells at 72 h post-treatment (Fig. 2B). Increased expression of crucial enzymes required for pyrimidine metabolism (dCTP deaminase Dcd), mRNA translation (leucine-tRNA ligase LeuS and lysine-tRNA ligase LysS), and cell wall biosynthesis (myo-inositol-1-phosphate synthase Ino1, methoxy mycolic acid synthase 1 MmaA1, probable acyl-[acyl-carrier protein] desaturase DesA1, probable fatty-acid-CoA ligase FadD22, fatty-acid-CoA ligase FadD28, and phenolpthiocerol synthesis type-I polyketide synthase PpsB) may promote mycobacterial replication, infectivity and pathogenicity. Particularly, the elevation of PpsB and FadD28 may elevate the production of phenolpthiocerol and phthiocerol dimycocerosate (dim), a major lipid virulence factor that modifies physical properties of host cell membranes to promote mycobacterial invasion and allow bacilli to escape from host immune responses [4, 5]. Likewise, the increase of FadD22 may lead to the rise of phenolic glycolipids (PGLs) which exert anti-bactericidal activity by inhibiting the production of nitric oxide in mycobacteria-infected macrophages [35], while a marked elevation of MmaA1 may induce the biosynthesis of mycolic acid, a critical determinant of mycobacteria-host interactions [43]. In addition, different essential virulence factors required for mycobacterial growth inside macrophages and mice were also induced by prolonged treatment of IFNγ, including histidine kinase KdpD of two-component regulatory system KDPD/KDPE, crucial machinery sensing environmental signals; mutator protein MutT2 responsible for removing an oxidatively damaged form of guanine from DNA and the nucleotide pool; and amidase AmiD involving in cellular metabolism [53, 54]. These findings suggest that IFNγ can modulate molecular signatures associated with proliferation and virulence in pathogenic mycobacteria via a direct interaction. On the other hand, the interaction with IFNγ also changed the expression of numerous hypothetical proteins; therefore, further functional studies of these uncharacterized proteins may not only elucidate the IFNγ-mycobacteria interaction but also reveal unrecognized virulence strategies of pathogenic mycobacteria.

### Secretion of mycobacterial extracellular vesicles is induced by IFNγ

In addition to identifying IFNγ-dependent molecular changes in mycobacteria by proteomics, we investigated the possible morphological changes of mycobacteria induced by IFNγ. *M. bovis* BCG was incubated with IFNγ or BSA for 72 h and analyzed by transmission electron microscopy (TEM). TEM analysis revealed that IFNγ treatment did not influence the size, shape, and cell envelop structure of *M. bovis* BCG; however, it triggered the secretion of extracellular vesicles (E.V.s) markedly; numerous membrane vesicles of various sizes, tens to hundreds nanometers in diameter, were found surrounding the IFNγ-treated mycobacteria whereas very few or no membrane vesicles were found in the BSA-treated bugs (Fig. 2C). Extracellular vesicles are critical elements in mycobacterial pathogenesis. They are membrane-bound structures carrying a variety of macromolecules and are released to promote environmental and host adaptation of mycobacteria [40, 46, 64]. Therefore, these data suggest that IFNγ promotes mycobacteria to a virulent phenotype.

### Interferon-γ promotes mycobacterial growth in culture

Findings from proteomic and TEM analyses suggest that interaction with IFNγ may impact mycobacterial growth and infection. We first evaluated the in vitro growth of *M. bovis* BCG in the presence of different concentrations of IFNγ, TNFα, IL1β, and IL12 by the colony-forming unit (CFU) method. Interferon-γ was found to enhance *M. bovis* BCG proliferation in a dose-dependent manner, whereas no other cytokines affected bacterial growth (Fig. 2D). In addition, we treated *M. smegmatis*, *M. avium*, and *S. typhimurium* with IFNγ, and analyzed their growth using the CFU method. We found that IFNγ supplementation did not alter the growth of *M. smegmatis* (Fig. 2E), *M. avium* (Fig. S1A) and *S. typhimurium* (Fig. S1B) at any concentration, which was possibly due to IFNγ does not interact with these bacterial species as shown in Fig. 1C. These data indicate that interaction with IFNγ promotes *M. bovis* BCG growth in vitro.

### Interaction with host IFNγ boosts intracellular growth of pathogenic mycobacteria in macrophages

**Fig. 3:**
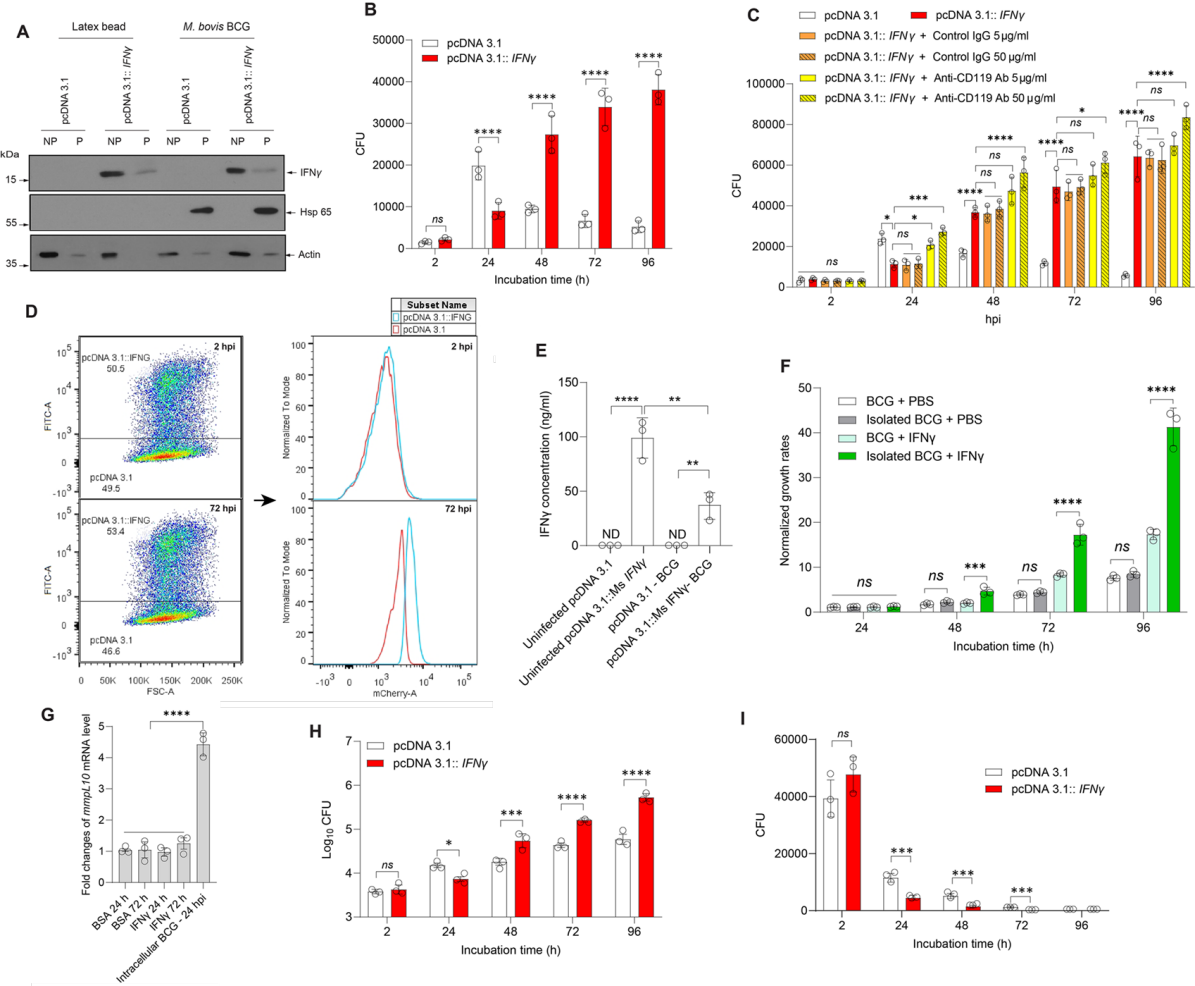
Interferon-γ promotes the intracellular growth of *M. bovis* BCG and *M. tuberculosis* in macrophages. (**A**) Exposure of internalized mycobacteria to IFNγ inside phagosome. Control or IFNγ-expressing macrophages were treated with 0.8 μm latex bead for 4 h infected with *M. bovis* BCG for 1 h followed by a 24 h chase and subjected to subcellular fractionation followed by immunoblotting with antibodies against IFNγ, Hsp65, and actin. Hsp65 and actin were used as control markers for fraction purity. N.P., non-phagosome fraction; P, phagosomal fraction. (**B**) Interferon-γ promotes intracellular growth of mycobacteria in macrophages. Control, IFNγ-expressing macrophages were infected with *M. bovis* BCG for 1 h, followed by chase for the indicated times before lysis and determination of CFU. (**C**) Blocking host IFNγ signaling enhances IFNγ-dependent mycobacterial growth in macrophages. Control or IFNγ-expressing macrophages were infected with *M. bovis* BCG for 1 h followed by chase for the indicated times. Antibodies against IFNγ receptor 1 (CD119) or control isotype were concurrently treated with infection. Intracellular growth of mycobacteria was evaluated by CFU assay. (**D**) Chimera assay demonstrated that IFNγ promotes intracellular growth of mycobacteria in macrophages. Control and IFNγ-expressing macrophages were mixed at a 1:1 ratio and infected with *M. bovis* BCG-mCherry for 1 h. At 2 and 72 h post-infection, cells were fixed, blocked, immunostained with FITC anti-IFNγ antibody, and analyzed by flow cytometry. (**E**) Interferon-γ production by interferon-γ-expressing macrophages. Control or interferon-γ-expressing macrophages were infected with *M. bovis* BCG for 1 h followed by a 48 h chase. Interferon-γ released to culture supernatant was quantified by sandwich ELISA. (**F**) Intracellular mycobacteria are more susceptible to IFNγ than cultured mycobacteria. Cultured or isolated intracellular *M. bovis* BCG was cultured in 7H9 broth without or with 10 µg/ml IFNγ supplementation. At the indicated times, the inoculum was serially diluted in PBS, plated on 7H10 agar plates, and incubated at 37^o^C for 3–4 weeks. The normalized growth rates were calculated as CFU values at indicated time points divided by their initial CFU values. (**G**) Host immune responses upregulate mycobacterial *mmpL10* expression. *M. bovis* BCG was isolated from macrophages at 24 h post-infection or collected from cultures without or supplemented with 10 µg/ml IFNγ for 24 and 72 h, and subjected to RNA extraction. Expression of the *mmpL10* gene was analyzed by qRT-PCR assay and the housekeeping *rpsO* transcript was used for normalization. (**H** and **I**) Interferon-γ promotes intracellular growth of *M. tuberculosis* but not *M. smegmatis* in macrophages. Control or IFNγ-rexpressing macrophages were infected with *M. tuberculosis* (**H**) or *M. smegmatis* (**I**) for 1 h, followed by chase for the indicated times before lysis and determination of CFU after 3 weeks (**H**) or 4 days (**I**) of incubation at 37^o^C. Data information: Statistical analyses in Fig. 3B, C, F and H, and 3I were performed with two-way ANOVA followed by multiple comparisons among groups, whereas statistical analysis in Fig. 3E and G was performed with one-way ANOVA followed by multiple comparisons among groups. The results are the mean values ± standard deviations of three biological replicates each with three (Fig. 3B, C, E, F and H, and 3I) or two (Fig. 3G) technical replicates. *, *p* < 0.05; **, *p* < 0.01; ***, *p* < 0.001; ****, *p* < 0.0001; *ns*, not significant. *, *p* < 0.05; **, *p* < 0.01; ***, *p* < 0.001; ****, *p* < 0.0001; ns, not significant

Previous studies have demonstrated IFNγ production at transcriptional and translational levels by various types of mouse and human macrophages [8] upon stimulation of IL12 and IL18 combination [6, 14, 33], IFNγ per se [17], U.V. exposure [66] or infections of *Salmonella typhimurium* [24, 38], *Legionella pneumophila* [52] and *Chlamydia pneumoniae* [21, 47–49, 55]. Interestingly, the combination of IL12 and infection of *M. bovis* BCG or *M. tuberculosis* is capable of inducing IFNγ production in human and mouse alveolar macrophages (AM), the primary conduit of infection [19, 44, 63], suggesting pathogenic mycobacteria may sense IFNγ to facilitate their intracellular growth in macrophages. To test this hypothesis, we infected control or IFNγ-expressing macrophages with *M bovis* BCG for 24 h and analyzed the intracellular localization of IFNγ using subcellular fractionation followed by immunoblotting. Cells treated with 0.8 μm latex bead for 4 h were used as controls. Mycobacterial HSP65 was detected only in the phagosomal fraction, suggesting that *M. bovis* BCG resides primarily in the phagosomes during the first 24 h after infection, while IFNγ was found predominantly in the cytosolic fraction but also in the phagosomal fraction where mycobacteria were present (Fig. 3A), suggesting a possible interaction of mycobacteria with IFNγ inside phagosomes. In addition, IFNγ was also detected in latex bead-containing phagosomes, suggesting that the translocation of IFNγ to phagosomes is solely regulated by host macrophages, not by pathogens. Interferon-γ production by macrophages is a poorly characterized phenomenon; therefore, the mechanism of IFNγ translocation to phagosomes remains to be discovered. However, previous studies of activated T._H_. cells, natural killer cells and eosinophils suggest that IFNγ is likely secreted through the conventional protein secretion (CPS) pathway [12, 23, 60]; IFNγ is transported from the endoplasmic reticulum to the Golgi apparatus and subsequently to the plasma membrane via secretory vesicles or secretory granules, implying that IFNγ may also be delivered to phagosomes, plasma membrane-bound vacuoles by a secretory compartment. However, further studies using a combination of molecular, biochemical and biophysical techniques are needed to elucidate the intracellular trafficking mechanism of IFNγ in macrophages.

We next infected control or IFNγ-expressing macrophages with *M. bovis* BCG and analyzed mycobacterial growth in macrophages using the CFU method. In control macrophages, mycobacteria were internalized and proliferated at first 24 h post-infection but were then eliminated by macrophages at later time points, while IFNγ expression reduced mycobacterial burden at 24 h post-infection but markedly increased mycobacterial growth at later time points compared with control cells (Fig. 3B), suggesting that IFNγ can induce the growth of intracellular mycobacteria similar to cultured mycobacteria (Fig. 2D). However, IFNγ was exported to the extracellular matrix once produced (Fig. S2), where it can bind to the IFNγ receptor 1 (CD119) to trigger mycobactericidal activities in macrophages. Therefore, to define whether the outgrowth phenotype of mycobacteria in IFNγ-expressing macrophages is due to a host-mediated impact of IFNγ or to a direct effect of IFNγ per se, we supplemented antibodies against CD119 to intercept the host IFNγ signaling (Fig. S3) following infection of control or IFNγ-expressing macrophages with *M. bovis* BCG and then analyzed the intracellular growth of mycobacteria. We found that blocking IFNγ signaling did not influence the intracellular growth of mycobacteria in control macrophages, which do not produce IFNγ (Fig. S2), but significantly enhanced mycobacterial growth in IFNγ-expressing cells in a dose-dependent manner (Fig. 3C), suggesting the mycobacterial outgrowth phenotype in IFNγ-expressing macrophages in Fig. 3B is due to a direct effect of IFNγ.

To independently examine the direct effect of IFNγ on the intracellular mycobacterial growth, we mixed equivalent amounts of control and IFNγ-expressing macrophages, infected with mCherry-*M. bovis* BCG, and analyzed bacterial burden in each cell type by flow cytometry. At 2 h post-infection, the bacterial load in the two cell types was similar, while at 72 h post-infection, the bacterial burden in IFNγ-expressing macrophages was significantly higher than that in the control cells (Fig. 3D), indicating intracellular IFNγ directly boost mycobacterial growth in macrophages. Interestingly, the concentrations of IFNγ that enhanced intracellular growth of mycobacteria in macrophages (Fig. 3E) were comparable to those produced by activated human T cells [10], human PBMC stimulated by *M. tuberculosis* antigens or *M. tuberculosis* [56, 61], or detected in lung homogenates of *M. tuberculosis*-infected mice [50], and were remarkably lower than the concentrations that promote mycobacterial growth in culture (Fig. 2D), suggesting that intracellular mycobacteria may be more sensitive to IFNγ than cultured mycobacteria. Therefore, to test this assumption, we isolated *M. bovis* BCG from macrophages at 24 h post-infection, cultured in the culture broth, treated with IFNγ, and analyzed its growth. We found that isolated and cultured mycobacteria grew similarly in cultures without IFNγ treatment; however, IFNγ promoted the growth of isolated mycobacteria one day prior cultured mycobacteria, and the IFNγ-induced growth increase was more remarkable in isolated mycobacteria than in cultured mycobacteria (Fig. 3F). In addition, the expression level of *mmpL10* was elevated in intracellular mycobacteria compared with cultured mycobacteria, and IFNγ treatment did not influence *mmpL10* expression (Fig. 3G). These data suggest that exposure to the host intracellular environment sensitizes mycobacteria to IFNγ, thereby allowing them to enhance intracellular growth.

To further validate the impact of IFNγ sensing mechanism on mycobacterial infection, we infected control or IFNγ-expressing macrophages with *M. tuberculosis* and non-pathogenic *M. smegmatis*, and then analyzed their intracellular growth. Similar to *M. bovis* BCG (Fig. 3B), *M. tuberculosis* was found to readily proliferate within macrophages, and the ability to sense IFNγ (Fig. 1C) allowed it to boost intracellular growth in macrophages (Fig. 3H). In contrast, *M. smegmatis* was rapidly cleared by macrophages, and expression of IFNγ further enhanced this clearance perhaps by enhancing the anti-bacterial effects (Fig. 3I).

Our findings clearly indicate that sensing host IFNγ is a crucial virulent strategy of pathogenic mycobacteria to promote their intracellular growth in macrophages.

### Interferon-γ-dependent induction of PpsB and AmiD promotes M. Tuberculosis growth in macrophages

**Fig. 4:**
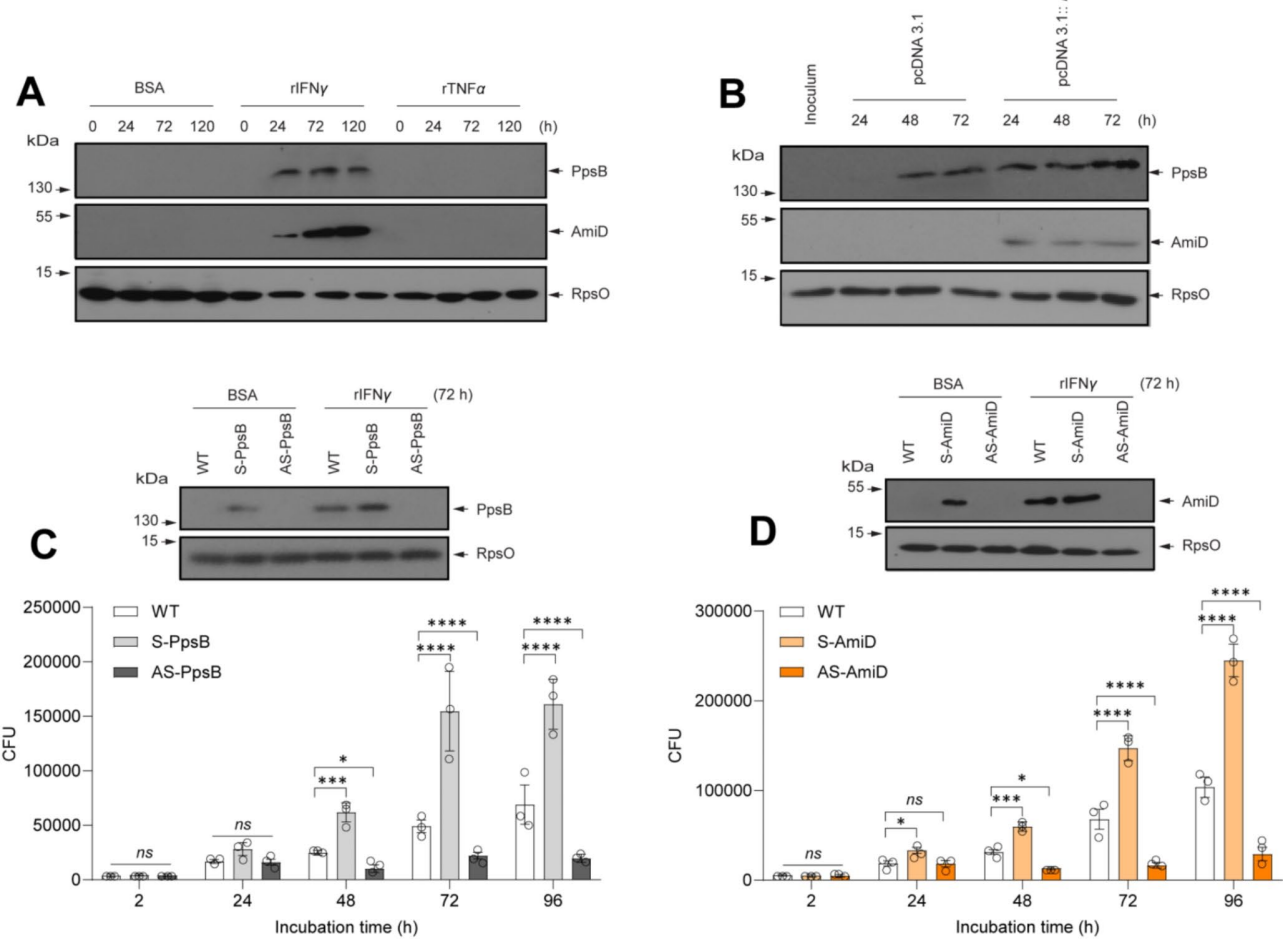
Induction of phenolpthiocerol synthesis type-I polyketide synthase (PpsB) and probable amidase (AmiD) expression by IFNγ promotes the growth of *M. tuberculosis* in macrophages. (**A**) Interferon-γ induces *M. tuberculosis* PpsB and AmiD expression in-vitro. Mycobacteria were cultured in 7H9 broth supplemented with 10 µg/ml BSA, IFNγ, or rTNFα. At indicated time points, bacteria were washed and analyzed by immunoblotting with antibodies against PpsB, AmiD, and RpsO. (**B**) PpsB and AmiD are expressed during *M. tuberculosis* infection in macrophages. Control or IFNγ-expressing macrophages were infected with *M. tuberculosis* for 1 h. The intracellular mycobacteria were isolated from infected macrophages at indicated time points and analyzed by immunoblotting with antibodies against PpsB, AmiD and RpsO. (**C**) Interferon-γ-induced PpsB expression promotes intracellular growth of *M. tuberculosis* in macrophages. Wild-type *M. tuberculosis*, *M. tuberculosis* transformed with pMV261-S-*pspB* (overexpression, S-PpsB), and *M. tuberculosis* transformed with pMV261-AS-*pspB* (knockdown, AS-PpsB) were cultured in 7H9 broth supplemented with 10 µg/ml BSA or IFNγ for 72 h before lysed and analyzed by immunoblotting with antibodies against PpsB and RpsO (Upper panel). Macrophages were infected with wild-type, S-PpsB, or AS-PpsB *M. tuberculosis* for 1 h followed by chase for the indicated times before lysis and determination of CFU (lower panel). (**D**) Interferon-γ-induced AmiD expression promotes intracellular growth of *M. tuberculosis* in macrophages. AmiD-overexpressing (S-AmiD) and AmiD-knockdown (AS-AmiD) *M. tuberculosis* were generated, characterized (upper panel) and analyzed their intracellular growth in macrophages (lower panel) as same as the procedure described in Fig. 4C. Data information: Statistical analyses in Fig. 4C and D were performed with two-way ANOVA followed by multiple comparisons among groups. The results are the mean values ± standard deviations of three biological replicates each with three technical replicates. *, *p* < 0.05; **, *p* < 0.01; ***, *p* < 0.001; ****, *p* < 0.0001; *ns*, not significant

To investigate the molecular mechanism via which IFNγ facilitates *M. tuberculosis* growth in macrophages, we focused on mycobacterial proteins that are induced by IFNγ (Table 1, Dataset 1). Phenolpthiocerol synthesis type-I polyketide synthase (PpsB) and amidase (AmiD) were most induced by IFNγ in *M. bovis* BCG, therefore, we examined whether IFNγ could directly modulate the expression of their homologs, PpsB (Rv2932) and AmiD (Rv3375) in M. tuberculosis. We cultured *M. tuberculosis* in the presence of 10 µg/ml BSA, IFNγ or TNFα, and analyzed the expression of PpsB and AmiD at different time points by immunoblotting. We were unable to detect PpsB and AmiD at their physiological levels, however, treatment of IFNγ but not TNFα markedly elevated the expression of PpsB and AmiD in *M. tuberculosis* (Fig. 4A).

We next analyzed PpsB and AmiD expression in the context of infection. We infected control or IFNγ-expressing macrophages with *M. tuberculosis*, and intracellular bacteria were isolated from macrophages at different time points for immunoblot analysis. Mycobacteria isolated from control macrophages displayed a higher expression level of PpsB but not AmiD at 48 and 72 h post-infection compared with cultured bacteria, whereas mycobacteria isolated from IFNγ-expressing cells showed increased expressions of both proteins at all time points compared with cultured bacteria (Fig. 4B), suggesting intracellular IFNγ is capable of triggering PpsB and AmiD expression in *M. tuberculosis* within macrophages.

The marked induction of mycobacterial PpsB and AmiD by IFNγ in culture and within macrophages suggests the potential importance of PpsB and AmiD in *M. tuberculosis* infection that remains unknown. Therefore, we generated *ppsB*-overexpressing (S-PpsB), *ppsB*-knockdown (AS-PpsB), *amiD*-overexpressing (S-AmiD) and *amiD*-knockdown (AS-AmiD) *M. tuberculosis* strains, characterized (Fig. 4, C and D; upper panel), infected them to macrophages, and analyzed their intracellular growth using the CFU method. We found that overexpression of *ppsB* or *amiD* gene enhanced intracellular *M. tuberculosis* growth while knockdown of *ppsB* or *amiD* gene dampened intracellular *M. tuberculosis* growth (Fig. 4, C and D; lower panel), indicating that *ppsB* and *amiD* are essential virulence factors of *M. tuberculosis* although *amiD* is expressed at a negligible level under physiological condition and during infection (Fig. 4, A and B).

Taken together, our findings suggest that IFNγ-dependent induction of PpsB and AmiD is an important virulence mechanism of *M. tuberculosis* to promote its growth in macrophages.

## Discussion

Interferon-γ is an indispensable immune effector, activating a robust protective-immune response against mycobacterial infection [20, 29], however, similar to *P. aeruginosa* which recognizes host cell activation through sensing IFNγ [65], pathogenic mycobacteria can also sense IFNγ (Fig. 1) to advance their pathogenesis. Direct interaction with IFNγ triggers mycobacteria to a virulence phenotype, secretion of extracellular vesicles and upregulation of numerous virulence factors (Fig. 2, B and C), thereby promoting mycobacterial growth in culture (Fig. 2D) and in macrophages (Fig. 3B).

Macrophages are essential effectors acting on the front-line of host defense against mycobacterial infection. Upon activation of IFNγ, macrophages exhibit a potent capacity to kill internalized mycobacteria [20]. However, the ability of pathogenic mycobacteria to survive in naïve and IFNγ-activated macrophages, and growing evidence that macrophages can produce IFNγ in response to various stimuli, including mycobacterial infection [19, 44, 63], suggest that sensing host IFNγ may be an important survival mechanism of pathogenic mycobacteria within macrophages. Although we showed the interaction of pathogenic mycobacteria with IFNγ in culture (Fig. 1, A-C); the binding of IFNγ from intracellular mycobacteria remains biochemically unclear due to technical limitations. However, corroborating the observations in cultured mycobacteria (Fig. 2D), exposure to IFNγ in phagosomes (Fig. 3A) leads to a marked increase of mycobacterial growth in macrophages (Fig. 3, B and D) that occurs independently of host IFNγ signaling (Fig. 3C), suggesting that intracellular mycobacteria perhaps sense host IFNγ to boost their growth in macrophages. Inhibition of IFNγ signaling elevated mycobacterial growth in IFNγ-expressing macrophages (Fig. 3C) and IFNγ-expressing macrophages produced significantly higher NO than control cells (Fig. S3), indicating that autocrine signaling of IFNγ exerts anti-mycobacterial effects in these cells. However, the significant increase of mycobacterial growth induced by intracellular IFNγ (Fig. 3, B and H) suggests that macrophage-derived IFNγ is more beneficial to mycobacterial infection than host immunity. Furthermore, cultured *M. bovis* BCG only senses IFNγ at concentrations above 1 µg/ml (Fig. 2D), and binding capacity of IFNγ to cultured *M. tuberculosis* is greater than that of *M. bovis* BCG (Fig. 1C), therefore in the previous study [2] the authors observed only the interaction of *M. tuberculosis* but not *B. bovis* BCG with IFNγ at concentrations less than 1 µg/ml. However, the host intracellular environment sensitizes mycobacteria to IFNγ through enhancing expression of *mmpL10* (Fig. 3G), thereby allowing intracellular mycobacteria to sense IFNγ at near-physiological concentrations (Fig. 3, B, E and H) [10, 50, 56, 61] approximately 200 times lower than that perceived by cultured mycobacteria (Fig. 2D). Although the ability of intramacrophage mycobacteria to sense IFNγ at near-physiological concentrations suggests the potential importance of this virulence mechanism in mycobacterial infection in human, further studies identifying host factor(s) that impact *mmpL10* expression are needed to enhance our understanding of how mycobacteria are transformed into a virulence phenotype in host macrophages.

Proteomics analysis reveals that multiple molecular signatures associated with mycobacterial proliferation and virulence are induced by IFNγ, among which AmiD, PpsB, and Dcd are the most induced but not fully characterized proteins (Table 1, Dataset 1). Besides being required for optimal growth of *M. tuberculosis* in mouse spleen [54], AmiD was identified as one of the culture filtrate proteins elevated most in prevalent *M. tuberculosis* strains compared with the laboratory strain *M. tuberculosis* H37Rv [26] and the target of HPOX, a new promising inhibitor against *M. tuberculosis* [34]. On the other hand, *ppsB* was found to be upregulated greater than 10-fold in rifampicin-resistant mutant *M. tuberculosis* relative to wild-type strain during infection of activated macrophages [7]. These studies suggest that *ppsB* and *amiD* could be important downstream targets of IFNγ in mycobacteria. Therefore, after uncovering that IFNγ markedly induced PpsB and AmiD expression in cultured and intracellular *M. tuberculosis* (Fig. 4, A and B), we evaluated the contributions of PpsB and AmiD to *M. tuberculosis* infection in macrophages. Imitating IFNγ’s effect, overexpression of *ppsB* or *amiD* significantly enhanced intracellular mycobacterial growth, whereas inhibition of *ppsB* or *amiD* expression diminished intracellular mycobacterial growth (Fig. 4, C and D; lower panel), indicating *ppsB* and *amiD* are not only important virulence factors but are also important mediators of IFNγ-dependent *M. tuberculosis* infection in macrophages. In addition to PpsB and AmiD, IFNγ also induces numerous other essential metabolism-related proteins (Fig. 2, A and B, Table 1), suggesting that the interaction with IFNγ can alter metabolic profile in mycobacteria. Therefore, identifying the IFNγ-induced metabolic signatures in mycobacteria could advance our understanding of how mycobacteria enhance their survival and growth in the host cells. On the other hand, the previous studies have shown that *dcd* homolog in *M. marinum* is specifically expressed in host granulomas but not in cultured macrophages [42], and the elevation of IFN-γ leads to an exacerbation of *M. tuberculosis* infection in mice [50] and tuberculosis reactivation in human [27], suggesting that IFNγ-dependent outgrowth of mycobacteria may be an unknown mechanism of tuberculosis activation and Dcd plays a central role in this mechanism. Therefore, further functional investigations of Dcd may uncover long-standing mysteries of tuberculosis reactivation. Interestingly, the induction of PpsB and AmiD by IFNγ was consistently observed in both cultured *M. bovis* BCG (Fig. 2B) and *M. tuberculosis* (Fig. 4A), and in intramacrophage *M. tuberculosis* (Fig. 4B), suggesting that the interaction with IFNγ relies on the bacterial state, particularly *mmpL10* expression level, however the signaling pathways activated by IFNγ may be highly conserved in *M. tuberculosis* complex and independent of IFNγ concentrations.

In addition to inducing anti-mycobacterial effects in macrophages; IFNγ can also trigger macrophages to express its own gene [17], raising certain concerns about the contribution of the self-activation mechanism of macrophages to host-mycobacteria interactions. Our findings indicate that the self-activation mechanism may provide an unrecognized niche for the mycobacterial infection as the bacilli can sense intracellular IFNγ to promote their growth within macrophages, therefore providing a typical example of the coevolution of pathogenic mycobacteria with their host macrophages. Interferon-γ induces outgrowth of intracellular mycobacteria by increasing their proliferative activity and activating multiple virulence mechanisms that enable mycobacteria to resist bactericidal effects and adapt to harsh intracellular environment inside the host cells. Therefore, this study does not only represent a hitherto unknown virulence strategy of pathogenic mycobacteria during infection, but may also pave the way for therapeutic approaches to treat mycobacterial diseases.

## Materials and methods

### Reagents

Antibodies and reagents used in this study were from the following sources: mouse anti-PpsB, anti-AmiD, and anti-RpsO antibodies were generated by injection of full-length recombinant *M. tuberculosis* PpsB, AmiD, or RpsO (100 µg) in 100 µl incomplete Freund’s adjuvant (Sigma, #F5506) to BALB/c mice on weeks 0, 2 and 5. Ten days after the final injection, serum samples were obtained, and ELISA was performed to determine the antibody titer; recombinant mouse IFNγ protein (#ab259378), recombinant mouse IL12 protein (#ab259419), recombinant mouse IL1β protein (#ab259421), recombinant mouse TNFα protein (#ab259411), and TMB ELISA substrate (#ab171523) from Abcam; mouse monoclonal anti-β-actin antibody (#3700S) from Cell Signaling; recombinant mouse IFNγ protein (#IF005) and glass bead (#G8772) from Sigma; recombinant mouse IL1β protein (#BMS332), recombinant mouse IL18 protein (#PMC0184), IFNγ rabbit monoclonal antibody (#701121), IFNγ monoclonal antibody (clone XMG1.2), eBioscience (#14-7311-81), rat anti-mouse IL12 p70 (clone 9A5) (#ENMM120), rabbit anti-mouse IL1β (#500-P51), rabbit anti-mouse IL18 (#210-401-323 S), rat IgG1 kappa isotype control (eBRG1), eBioscience (#14-4301-82), goat anti-rabbit IgG-HRP (#31460), goat anti-rat IgG-HRP (#31470), geneticin selective antibiotic (G418 Sulfate) (#10131035), and fluoromount-G mounting medium (#00-4958-02) from Thermo Fisher Scientific; FITC anti-mouse IFNγ antibody (#505806), FITC goat anti-rat IgG antibody (#405404), and Alexa fluor 647 anti-mouse IgG1 antibody (#406618) from BioLegend; rat anti-mouse IFNγ receptor 1 (CD119) (clone GR-20) and rat IgG2a isotype control (clone 2A3) from BioXCell; and heat shock protein 65 (mycobacterial) monoclonal antibody (clone 4H11) (#ADI-SPA-882-E) from Enzo Life Science.

### Bacterial and cell culture

*M. tuberculosis* H37Rv (ATCC, #25618), *M. bovis* BCG Pasteur (ATCC, #35734), *M. avium* (ATCC, #25291), *M. smegmatis* mc2 155 (ATCC, #700084) were cultured in 7H9 media (Fisher Scientific, #DF0713-17-9) supplemented with 10% OADC enrichment (Fisher Scientific, #B12351) while *S. typhimurium* (ATCC, #14028) was cultured in L.B. media at 37 ^o^C until the stationary phase. *M. tuberculosis* H37Rv was cultured at a biosafety laboratory level 3 containment facility. *M. bovis* BCG-mCherry was generated by transforming pMSP12::mCherry to *M. bovis* BCG. The pMSP12::mCherry plasmid was a gift from Lalita Ramakrishnan (Addgene plasmid #30169; http://n2t.net/addgene:30169; RRID: Addgene_30169).

Mouse macrophages RAW 264.7 (ATCC, #TIB-71) were grown in RPMI 1640 (Thermo Fisher Scientific, #11875093) supplemented with 10% FBS (Thermo Fisher Scientific, #26140079), and 1% penicillin/streptomycin (Gibco, #15140122). Cells were incubated in a 37 oC humidified incubator with 5% CO2.

### Gene overexpression and knockdown in M. Tuberculosis

Overexpression and knockdown of *ppsB* or *amiD* in *M. tuberculosis* were performed as previously described [59]. Briefly, the *ppsB* (4,617 bp) or *amiD* (1,429 bp) genes were amplified by PCR using the primer pairs (PpsB-forward primer, CCG AAG CTT GTG ATG CGA ACG GCT TTC AGC and PspB-reverse primer, CCG AAG CTT TCA TTG TGT TCC TCT TAG TCG; AmiD-forward primer, CCG AAG CTT ATG ACC GAT GCT GAC AGT GCG and AmiD-reverse primer, CCG AAG CTT TCA CAC CGG CGG GCG TCG GC; HindIII site underlined), and cloned into pMV261 (4,488 bp) cut at HindIII of the multi-cloning site. Positive clones were digestion checked by PvuII. Overexpression clones (sense orientation) would give fragments of approximately 6.25 and 2.85 kbp (*ppsB*) or 5.75 and 0.17 kbp (*amiD*), while knockdown clones (anti-sense orientation) would give pieces of roughly 7.3 and 1.8 kbp (*ppsB*) or 4.64 and 1.27 kbp (*amiD*). The plasmids were then electroporated into competent *M. tuberculosis*, followed by immediate culture in an antibiotic-free 7H9 broth for 2 days and plating on 7H10 agar plates containing 25 µg/ml kanamycin.

### Generation of IFNγ-overexpressing macrophages

RAW 264.7 cells were transfected with the control pcDNA3.1 (Thermo Fisher Scientific, #V79020), or pcDNA3.1::Ms IFNγ (GenScript, #Omu18252) plasmids using Lipofectamine 3000 transfection reagent (Thermo Fisher Scientific, #L3000008). The cells were cultured in an antibiotic-free culture medium for 2 days, followed by a selection of 1.5 mg/ml G418 (Thermo Fisher Scientific, #10131027) for 12 days. The medium was replaced every 4 days. Production of interferon-γ was validated by immunoblotting and ELISA.

### Infection assay

Bacterial growth inside macrophages was evaluated by colony-forming unit (CFU) assay as previously described [62]. Briefly, control or IFNγ-expressing macrophages were infected with *M. bovis* BCG, *M. tuberculosis* or *M. smegmatis* at MOI of 10 for 1 h, followed by an incubation with culture medium containing Amikacin (200 µg/ml) for 2 h to kill extracellular bacteria, and re-cultured in antibiotic-free culture medium. At indicated times; cells were washed three times with PBS, lysed in ddH2O for 10 min at 37^o^C, serially diluted in PBS, and plated on 7H10 agar plates. Bacterial CFU was determined after 3–4 weeks (*M. bovis* BCG and *M. tuberculosis*) or 4 days (*M. smegmatis*) of incubation at 37 ^o^C.

For the blocking assay, anti-IFNγ receptor 1 (CD119) antibody or isotype control were concurrently treated with infection at concentrations of 5 and 50 µg/ml. Intracellular growth of mycobacteria was evaluated by the CFU assay as described above.

### Chimera assay

Wild-type and IFNγ-expressing macrophages were mixed at a 1:1 ratio 4 h before infection with *M. bovis* BCG-mCherry at MOI of 10 for 1 h, followed by incubation with culture medium containing Amikacin (200 µg/ml) for 2 h, and re-cultured in antibiotic-free culture medium. At indicated times, cells were washed with PBS, fixed with 4% paraformaldehyde at 37 ^o^C for 30 min, permeabilized in 0.2% saponin in PBS for 30 min at room temperature, and blocked with 2% goat serum in PBS (blocking buffer) at room temperature for 1 h. Cells were incubated with FITC anti-IFNγ antibody (1:200 dilution in blocking buffer) for 1 h at room temperature, washed with PBS, resuspended in 200 µl FACS buffer (PBS, 2% FCS, 0.1% NaN3, 5 mM EDTA), and analyzed by the BD FACS CantoII Analyzer. The results were analyzed with FlowJo software (Tree Star).

### Bacterial growth with cytokine supplementation

Bacteria were collected in the stationary phase, washed with PBS, adjusted to OD600 = 0.1 by appropriate media, and then cultured 5 ml of bacterial suspensions in 50 ml Falcon tubes with the addition of different concentrations of cytokines. At indicated times, 20 µl bacterial suspensions were collected, diluted in PBS, plated on 7H10 (mycobacteria) or L.B. (*Salmonella*) agar plates, and incubated in 37 ^o^C for 3–4 weeks (*M. bovis* BCG and *M. tuberculosis*), 4–6 days (*M. smegmatis*), 10–14 days (*M. avium*) or 1 day (*S. typhimurium*) for CFU determination.

To investigate the impact of intramacrophage environment on the response of mycobacteria to IFNγ, we inoculated nearly equal amounts of cultured *M. bovis* BCG (5.7 × 10^7^, 4.9 × 10^7^, and 7.6 × 10^7^ CFU in three biological replicates, respectively) and isolated intracellular *M. bovis* BCG (approximately 3.8 × 10^7^, 6.4 × 10^7^, and 6.1 × 10^7^ CFU in three biological replicates, respectively) in 2 ml of 7H9 media without or with 10 µg/ml IFNγ supplementation. At the indicated time points, the inoculum was serially diluted in PBS, plated on 7H10 agar plates, and incubated at 37 ^o^C for 3–4 weeks. The normalized growth rate was calculated as the CFU values divided by their initial CFU values. The above-mentioned amounts of cultured mycobacteria corresponded to 0.5 ml of stationary phase *M. bovis* BCG at OD600 of 0.1, whereas the above-mentioned amounts of intracellular mycobacteria were isolated from 10 150-mm macrophage dishes (2 × 10^7^ cells/dish) infected with *M. bovis* BCG at an MOI of 10 for 24 h.

### Cytokine quantification

The secretion of IFNγ from macrophages was analyzed by sandwich ELISA. Control or IFNγ-expressing macrophages were infected with *M. bovis* BCG for 1 h followed by a 48 h chase. IFNγ concentration in culture supernatant was analyzed using a mouse IFNγ quantikine ELISA kit (R&D Systems, #MIF00) according to the manufacturer’s instructions.

### ELISA binding assay

Binding of IFNγ to mycobacteria was evaluated as previously reported [65]. Briefly, *M. bovis* BCG harvested in the stationary phase was washed with PBS and fixed in 4% paraformandehyde for 30 min at room temperature. The fixed bacteria were adjusted to OD600 = 0.4 with PBS and coated in the MaxiSorp ELISA plate by carbonate-bicarbonate coating buffer (pH9.6) overnight. Nonspecific binding sites were blocked with 3% BSA for 1 h at room temperature, followed by the addition of varying concentrations of IFNγ, heat-inactivated IFNγ, BSA, IL12, IL1β or IL18, and incubated overnight at 4 ^o^C. Samples were washed and incubated with rat anti-mouse IFNγ (clone XMG1.2), rat anti-mouse IL12 (clone 9A5), rabbit anti-mouse IL1β, or rabbit anti-mouse IL18 at a dilution of 1:1,000 for 2 h at room temperature. Goat HRP-conjugated anti-rat or goat HRP-conjugated anti-rabbit antibody was added to the washed samples at 1:10,000 dilution and incubated for 1 h at room temperature. TMB was then added as a substrate and the reaction was read at 450 nm after the addition of stop solution.

### Fluorescence microscopy

*M. bovis* BCG harvested in the stationary phase was washed with PBS, fixed in 4% paraformandehyde for 30 min at room temperature, blocked with 3% BSA for 1 h at room temperature, and incubated with 10 µg BSA, IL12, IFNγ or heat-inactivated IFNγ overnight at 4 ^o^C. After washing, bacteria were stained with mouse anti-HSP65 and FITC rat anti-IFNγ or rat anti-IL12 at 1:50 dilution for 2 h at room temperature. Bacteria were washed and incubated with Alexa 674 rabbit anti-mouse IgG and/or FITC goat anti-rat IgG at 1:50 dilution for 1 h at room temperature. Bacteria were washed, fixed to the slide, mounted with Fluoromount-G mounting medium and analyzed using a laser scanning confocal microscope (Olympus FV1000, Japan) followed by image processing by the FV10-ASW Viewer 3.1 software.

### Transmission electron microscopy

*M. bovis* BCG was cultured in the presence of 10 µg/ml BSA or IFNγ for 72 h, collected, and then fixed in a fixative containing 2.5% glutaraldehyde and 3 mM CaCl3 in 0.1 M cacodylate buffer for 1 h at room temperature. After washing with 0.1 M cacodylate buffer containing 3 mM CaCl3, the cells were post-fixed for 1 h at 4 °C in 0.1 M cacodylate buffer containing 1% osmium tetroxide and 1.5% potassium ferricyanide. The samples were washed briefly in distilled water, followed by gradual dehydration in a graded ethanol series of 70%, 90%, 95% (15 min per stage), and then 100% (three times and 30 min each time). The dehydrated samples were then infiltrated in stages with spur resin–ethanol solutions containing 50%, 75%, and 100% resin (1 h per stage). The infiltrated samples were left in 100% spurr resin overnight. Next, samples were embedded in the models with fresh resin and polymerized at 70 °C for 24 h. The embedded cells were cut using an ultramicrotome (Ultracut S, Leica Reichart) into 70 nm ultrathin sections using a diamond knife and then post-stained with uranyl acetate and lead citrate. Transmission electron microscopy was carried out on a JEOL JEM-1400 at 120 keV equipped with CCD Camera System (Ultrascan, Gatan).

### LC-MS/MS analysis

*M. bovis* BCG was cultured in the presence of 10 µg/ml BSA, IFNγ or heat inactivated IFNγ for 24 and 72 h. Bacteria were collected, washed with PBS and TEN buffer (75 mM Tris-HCl pH8.8, 4 mM EDTA, 100 mM NaCl), and resuspended in TEN buffer. Bacterial suspension was mixed with glass bead and subjected to homogenization at 5,000 rpm using Minilys personal homogenizer (Bertin Corp, #P000673-MLYS0-A). After centrifugation at 12,000 xg for 10 min at 4 ^o^C, bacterial protein was collected as supernatant, quantified by BCA assay, mixed with 4 volumes of ice-cold acetone, and incubated overnight at -80 °C. Samples were pelleted by 15,000 g for 10 min at 4 °C). The pellet was washed in ice-cold acetone for three times followed by repeated pelleting. Protein pellets were dissolved in 6 M urea and a total of 20 µg proteins from each sample were used. Proteins were reduced by incubation with 10 mM dithiothreitol (DTT) for 1 h at 29 °C and alkylated by 55 mM iodoacetamide (IAA) for 1 h at room temperature in the dark. This step was quenched by 55 mM DTT for 45 min. Protein digestion was performed overnight at 37 °C using mass spectrometry grade modified trypsin (Promega) at a 1:50 trypsin/protein ratio. After overnight incubation, 0.1% TFA was added to stop the digestion. Finally, all remaining reagents from the in-solution digestion procedure were removed using C18 stage tip.

LC-MS/MS analysis was performed on a Orbitrap Fusion Lumos Tribrid quadrupole-ion trap-Orbitrap mass spectrometer (Thermo Fisher Scientific, San Jose, CA) equipped with a NanoSpray ion source. Peptides were separated on an Ultimate system 3000 nanoLC system (Thermo Fisher Scientific, Bremen, Germany). Peptide mixtures were loaded onto a 75 μm ID, 25 cm length C18 Acclaim PepMap NanoLC column (Thermo Scientific, San Jose, CA, USA) packed with 2 μm particles with a pore of 100 Å. Mobile phase A was 0.1% formic acid in water, and mobile phase B was composed of 100% acetonitrile with 0.1% formic acid. A segmented gradient in 90 min from 2 to 35% solvent B at a flow rate of 300 nl/min and a column temperature of 35 °C were used. Mass spectrometry analysis was performed in a data-dependent mode with Full-MS (externally calibrated to a mass accuracy of < 5 ppm, and a resolution of 120,000 at m/z = 200, AGC target 5e5, maximum injection time of 50 ms) followed by HCD-MS/MS of the most intense ions in 3 s. High-energy collision activated dissociation (HCD)-MS/MS was used to fragment multiply charged ions within a 1.4 Da isolation window at a normalized collision energy of 32. AGC target 5e4 was set for MS/MS analysis with previously selected ions dynamically excluded for 60 s. Max injection time of 50 ms. Ions were detected at Orbitrap with resolution 15,000.

The raw MS/MS data were searched against the UniProt knowledgebase reviewed *M. bovis* BCG Pasteur 1173P2 protein database (downloaded on April 2022) by using the Mascot search algorithm (version 2.3) via the Proteome Discoverer (PD) package (version 2.2, Thermo Scientific). The search parameters were set as follows: peptide mass tolerance, 10 ppm; MS/MS ion mass tolerance, 0.02 Da; enzyme set as trypsin and allowance of up to two missed cleavages; variable modifications included oxidation on methionine, deamidation on asparagine and glutamine residues, and carbamidomethylation of cysteine residues. Peptides were filtered based on a 1% FDR. For relative protein quantification across different samples, each protein group is represented by a single master protein (P.D. Grouping feature) and the raw abundance of each protein was normalized by total abundance [36]. Data are available via ProteomeXchange with identifier PXD038542. Protein expression fold change between two groups was calculated from the average of their three biological replicates. Proteins with at least 1.5 fold changes in IFNγ-treated bacteria but less than 1.5 fold changes in HI-IFNγ-treated bacteria compared with BSA control were selected and statistically analyzed. Proteins that were significantly changed by IFNγ but not by HI-IFNγ were listed in Table 1 and their abundance was presented by heatmap via http://www.heatmapper.ca/.

### Intracellular bacteria isolation and immunoblotting

Isolation of intracellular *M. tuberculosis* from infected macrophages was performed as previously described [31]. In brief, infected cells were washed with PBS and lysed in GTC lysis buffer (4 M guanidine thiocyanate, 0.5% sodium N-lauryl sarcosine, 25 mM tri-sodium citrate, 0.1 M 2-mercaptoethanol, 0.5% Tween-80, pH 7). Bacterial pellet was collected after centrifugation at 5,000 xg for 20 min, washed, and resuspended in TEN buffer. The glass bead was added to the bacterial suspension and subjected to homogenization at 5,000 rpm using Minilys personal homogenizer. After centrifugation at 12,000 xg for 10 min at 4 ^o^C, bacterial protein was collected as supernatant, quantified by BCA assay, and analyzed by immunoblotting with antibodies against PpsB, AmiD and RpsO.

### Quantitative RT-PCR

This assay was done as previously described [22, 45] with some modifications. Briefly, *M. bovis* BCG cultured in the presence of 10 µg/ml BSA or IFNγ was collected at 24 and 72 h post-treatment, whereas intracellular *M. bovis* BCG was isolated from infected macrophages at 24 h post-infection as reported before [31]. Total ribonucleic acid (RNA) from cultured or intracellular mycobacteria was isolated using the glass bead and TRIzol solution, and residual contaminating genomic DNA was degraded by TURBO DNase (Thermo Fisher Scientific, #AM2238). The complementary DNA (cDNA) was prepared with RevertAid RT reverse transcription kit (Thermo Fisher Scientific, #K1691) and the qRT-PCR was carried out using SYBR Green PCR master mix (Thermo Fisher Scientific, #4039155), primers (*mmpL10*, forward: 5’-CCG TGA TGT TGC TAG TCA TCT-3’ and reverse: 5’-GCT TGA TTG GAT ACG GCT AGA-3’; *rpsO*, forward: 5’-AAG GAG ATT CTG CGC TCC TA-3’ and reverse: 5’-CGA ATG ATG GTC GTG CTT GT-3’) and PCR program (95 ^o^C for 10 min, 40 cycles of 95 ^o^C for 15 s and 55 ^o^C for 1 min, increment 0.3 ^o^C to 95 ^o^C for melt curve). The housekeeping *rpsO* transcript was used for the normalization and the fold changes of transcript were calculated using the 2^−ΔΔCT^ method.

### Subcellular fractionation and immunoblotting

Subcellular fractionation was performed as previously described with some modifications [16, 57]. Briefly, macrophages were washed with cold PBS and homogenization buffer (250 mM sucrose, 3 mM imidazole, pH 7.4), harvested, and resuspended in cold homogenization buffer. Cells were homogenized through Dounce homogenizer, followed by centrifugation at 1,000 xg at 4oC for 5 min. The supernatant was collected, brought to 40% sucrose by adding an equal volume of 62% sucrose solution, and loaded onto the cushion of 62% sucrose. The 35, 25, and 10% sucrose solutions were added one after another, followed by centrifugation at 100,000 xg for 1 h at 4oC. The phagosomal fraction was collected at the interface of the 10 and 25% sucrose solutions while the non-phagosome fraction was a mixture of fractions collected at the interfaces beneath other sucrose gradients. The phagosomal fraction was resuspended in cold PBS and centrifuged at 40,000 xg for 15 min at 4oC. Phagosomes were collected as pellet and lysed in RIPA (50 mM Tris-HCl pH7.4, 150 mM NaCl, 1% NP-40, 0.5% Na deoxycholate (NaDOC), 0.1% SDS, 1mM EDTA) buffer containing halt protease and phosphatase inhibitor cocktail (Thermo Fisher Scientific, Cat# 1861284) in 30 min at 4oC. Protein solution obtained from the supernatant after centrifugation at 10,000 xg for 10 min at 4oC was subjected to subsequent immunoblotting. On the other hand, the non-phagosome fraction was subjected to trichloroacetic acid-mediated protein precipitation, washed with acetone, and centrifuged at 12,000 xg for 5 min at 4oC. The pellet was reconstituted in RIPA buffer containing halt protease and phosphatase inhibitor cocktail, incubated in 30 min at 4^o^C, and centrifuged at 10,000 xg for 10 min at 4^o^C. The supernatant was collected and used for subsequent immunoblotting analysis.

Phagosomal and non-phagosome fractions were quantified by BCA protein quantification (Thermo Fisher Scientific, #23225), separated on 10% SDS-PAGE gels, and transferred onto nitrocellulose membranes with semi-dry transfer systems (BioRad, Hercules, CA, USA). The membranes were blocked with blocking buffer (5% BSA in PBS-Tween20) overnight at 4 °C, followed by an incubation of primary antibodies diluted in blocking buffer at 1:2,000 for anti-actin antibody and 1:1,000 for other antibodies for 2 h at room temperature. The membranes were washes, incubated with horseradish peroxidase (HRP)-conjugated secondary antibody for 1 h at room temperature, developed and imaged.

### Statistical analyses

Statistical analyses were performed with one-way or two-way ANOVA followed by multiple comparisons among groups in Prism (GraphPad Software). The results are mean values ± standard deviations from three independent experiments unless otherwise defined in the legends. Error bars show standard deviation.

### Electronic supplementary material

Below is the link to the electronic supplementary material.

Supplementary Material 1

Supplementary Material 2

## Data Availability

The mass spectrometry proteomics data have been deposited to the ProteomeXchange Consortium via the PRIDE [37] partner repository with the dataset identifier PXD038542.
