# Supplementary material for: Enhancement of mycobacterial pathogenesis by host interferon-γ
Source: Cell Mol Life Sci. 2024 Sep 2;81(1):380. doi: 10.1007/s00018-024-05425-7 (PMC11368887; doi:10.1007/s00018-024-05425-7)
Supplement: Supplementary file 2 — Supplementary Material 2 [file 18_2024_5425_MOESM2_ESM.docx]

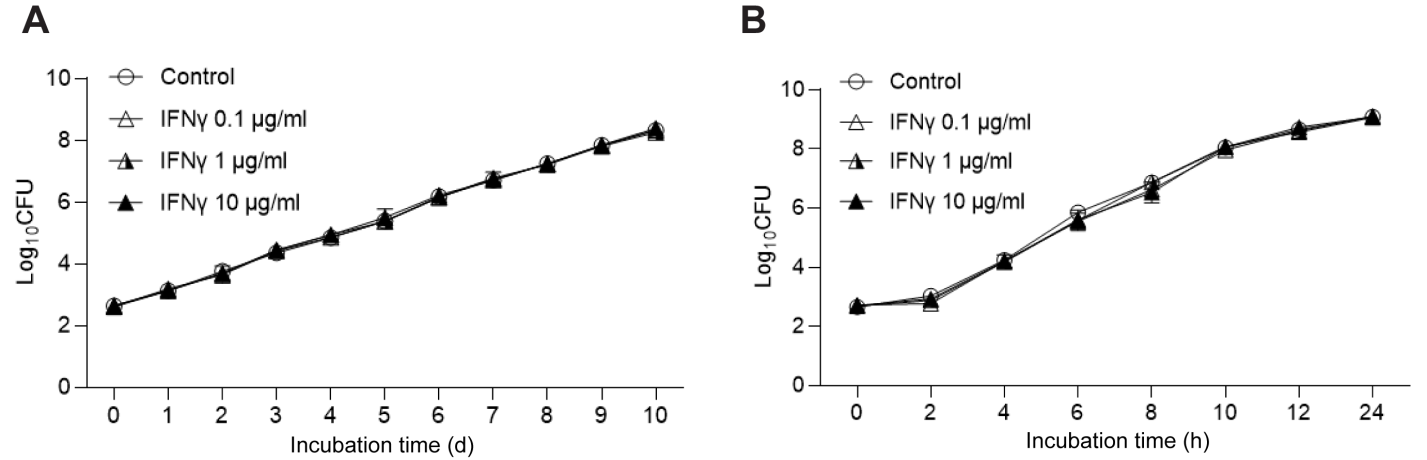


**Figure S1. Bacterial growth in the presence of IFNγ**

M. avium (A) and S. typhimurium (B) were cultured in 7H9 and L.B. broth, respectively, and supplemented with IFNγ at different concentrations. At indicated time points, cultures were serially diluted in PBS, and plated on 7H10 (M. avium) or L.B. (S. typhimurium) agar plates. The bacterial CFU was determined after 10-14 days (A) or 1 day (B) of incubation at 37^o^C. *, *p*<0.05; **, *p*<0.01; ***, *p*<0.001; ****, *p*<0.0001; ns, not significant.

**
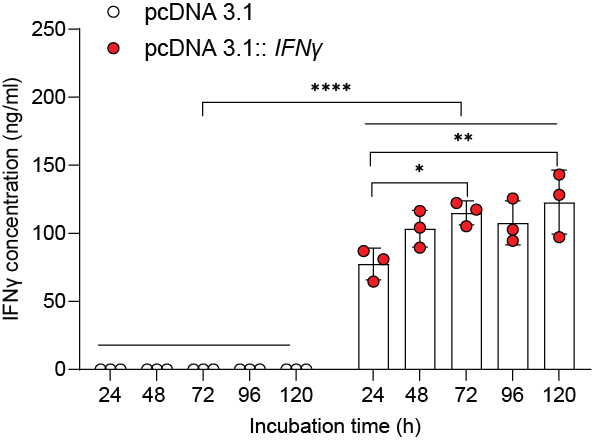
**

**Figure S2. Interferon-γ production by IFNγ-expressing macrophages**

Control or interferon-γ-expressing macrophages were cultured for 5 days. Interferon-γ released to culture supernatant was quantified by sandwich ELISA at the indicated time points. *, *p*<0.05; **, *p*<0.01; ***, *p*<0.001; ****, *p*<0.0001; ns, not significant.

**
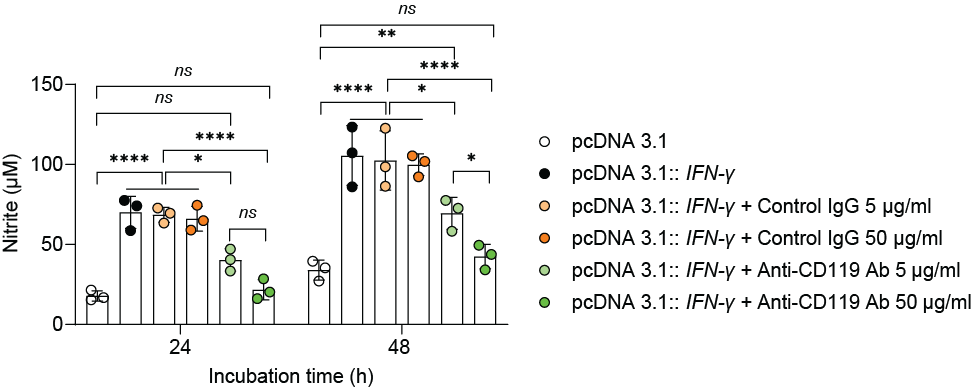
**

**Figure S3. Nitrite analysis showed effective inhibition of IFNγ signaling by anti-CD119 antibody**

Culture supernatant samples collected from *M. tuberculosis*-infected cells at 24 and 48 h post-infection in Fig. 3C were analyzed by Griess assay. *, *p*<0.05; **, *p*<0.01; ***, *p*<0.001; ****, *p*<0.0001; ns, not significant.
